# PICO™ (Closed-Incision Negative-Pressure Wound Therapy) Dressing Use as Postoperative Prophylaxis for Preventing Surgical Site Infections in Spinal Surgery: A Retrospective Single-Centre Study

**DOI:** 10.7759/cureus.69214

**Published:** 2024-09-11

**Authors:** Hassan Imtiaz, Chadi Ali, Hilali Noordeen, Hanny Anwar

**Affiliations:** 1 Trauma and Orthopaedics, Poole General Hospital, Poole, GBR; 2 Spine Surgery, Royal National Orthopaedic Hospital, London, GBR

**Keywords:** negative pressure wound therapy, pico, spinal surgery, surgical site infections, wound complication

## Abstract

Introduction

Surgical site infections (SSIs) are one of the dreaded complications of spinal surgery. These typically develop within the first 30 days following surgery. The overall pooled incidence of SSIs is reported at 3.1%. Negative pressure wound therapy (NPWT) has been employed for the management of open wounds and soft tissue injury. There has been a recent trend towards the use of closed incision NPWT (ciNPT), such as PICO. There are only a few studies evaluating the prophylactic use of ciNPT in spinal surgery. The aim of this study was to evaluate whether prophylactic use of PICO dressings can reduce SSI incidence and complications in spinal surgery.

Methods

Data were collected retrospectively for patients undergoing spinal surgery, with a PICO dressing used for closed surgical incisions, from February 2021 to October 2022. Each patient was followed up for 30 days. The results were compared with local hospital infection control statistics for previous years.

Results

A total of 50 patients underwent spinal surgery and had PICO dressings post-operatively. None of the patients developed a seroma. Two out of 50 (4.0%) patients developed wound dehiscence and then subsequent SSI (1 superficial, 1 deep). These were managed conservatively with the use of antibiotics and prolonged ciNPT. None of these patients returned to the theatre. The average SSI incidence from previous years was 9.27 ± 4.14 per annum (1.15%), but with an average of 77.3% of these requiring a return to theatres.

Conclusion

Our study reflects that there is no difference in the incidence rates for SSIs for patients who have PICO dressings versus those having standard occlusive dressings as post-operative closed surgical incision site wound closure following spinal surgery. For those who do develop SSIs, there was no difference in the rates of return to theatres among the two patient populations.

## Introduction

Surgical site infections (SSIs) are one of the most dreaded complications following spinal surgery. These typically develop within the first 30 days after surgery. The overall pooled incidence of SSIs is estimated at 3.1% (superficial SSI: 1.4%, deep SSI: 1.7%). Subgroup analysis shows the highest incidence of SSIs in patients undergoing surgery for neuromuscular scoliosis [1]. SSIs in neurosurgery (including spinal surgery) incur the highest costs of all specialty-based SSIs, with an average increased cost of $23,755 per case [2].

In 2022, the total number of spinal surgery procedures performed in the UK was 65,594 [3]. The UK Health Security Agency Surgical Site Infection Surveillance report highlighted that patients undergoing multiple procedures for spinal surgery increased from 1.0% to 6.2% [4]. NHS resolution data suggest that, between 2009 and 2019, the total estimated cost of closed claims related to spinal surgery was £100 million [5]. The large volume of spinal procedures being carried out annually, along with a rising trend of requiring multiple interventions and potential costs of litigation, emphasizes the impact that SSIs in this patient group can bear in terms of financial and healthcare burden.

Traditionally, occlusive dressings are used post-operatively for closed spinal surgery incisions. They provide a sterile barrier between the surgical site and the external environment, therefore reducing the chances of infiltration of microbes from the skin and the surrounding environment. Due to the high incidence of SSIs in spinal surgery patients, surgeons have tried a multitude of dressing types to minimize the risk of these complications. 

One of the commonly employed wound management dressing types is negative pressure wound therapy (NPWT). It has been employed for the management of open wounds and soft tissue injuries [6-8] and is commonly used to treat SSIs [9]. This usually involves the use of vacuum-assisted closure devices (VAC). It works on the principle of applying sub-atmospheric pressure to the wound, thereby clearing any exudate and promoting wound healing by means of inducing the development of granulation tissue. The available literature addresses the use of NPWT as a means of managing SSI, but there is scarce evidence for the use of NPWT as a prophylactic measure to prevent the incidence of SSI [10].

Closed incision NPWT (ciNPT) is becoming more popular for managing and promoting the healing of closed surgical incisions [11-14]. In addition to promoting wound healing, it provides a barrier for entry of pathogens from the surrounding environment into the surgical site due to the presence of negative pressure creating a seal around the wound margins.

A few studies have indicated that the use of ciNPT for spinal surgery is associated with a lower incidence of surgical site occurrences such as seroma, hematoma, or wound dehiscence [15], but have not indicated its significance in reducing the incidence of SSIs or any subsequent complications.

One such form of ciNPT is PICO, a portable negative pressure wound therapy system designed for single use, lasting seven days. It consists of a sterile pump with four layers of adhesive dressings. It is operated by a battery and can generate a pressure of up to 80 mmHg [16]. Our spinal department began the use of PICO dressings as post-operative prophylaxis for SSIs in spinal surgery patients in February 2021. The aim of this study was to evaluate the effectiveness of these particular NPWT dressings compared to standard occlusive dressings in reducing the incidence of SSI in spinal surgery patients postoperatively.

This article was previously presented as a poster presentation at the 2023 BritSpine Annual Scientific Meeting on April 18-20, 2023.

## Materials and methods

This study involved patients from a single centre. Data were collected retrospectively. We began the use of PICO dressing as postoperative surgical site infection (SSI) prophylaxis in February 2021. Therefore, we collected data for patients undergoing spinal surgery between February 2021 and October 2022. The measured outcomes for these patients were age, American Society of Anesthesiologists (ASA) grade, wound classification (clean, clean contaminated, contaminated, or dirty), spinal procedure, duration of PICO dressing application, and incidence of wound complications within 30 days post operation, along with their subsequent management. Wound complications consisted of seroma formation, wound dehiscence, and SSI. SSIs were further classified into superficial, deep, and those involving organ space.

The incidence of SSIs was compared among patients who had PICO dressings postoperatively with those who had routine wound dressings. We compared the infection rates from the last six years. These data were provided by the infection control department at our hospital.

For this study, we excluded patients who did not undergo spinal surgery and those who did not have PICO dressings. Patients with any other form of negative pressure wound therapy such as vacuum-assisted closure dressings were also excluded. SSIs occurring beyond 30 days following surgery were also excluded [17].

Data were collected onto an excel spreadsheet. Statistical analysis was performed using JASP (version 0.18.3; https://jasp-stats.org). Statistical significance was evaluated using a chi-squared test. A p value of less than 0.05 was considered statistically significant.

## Results

A total of 50 patients undergoing spinal surgery between February 2021 and October 2022 had PICO dressings used as postoperative SSI prophylaxis. The mean age was 32.7 years (range: 13-81 years). Additionally, 30/50 patients were males; 30/50 patients had an ASA grade of 1; and 16/50 had an ASA grade of 2, with the remaining 4/50 patients having an ASA grade of 3.

All surgical site wounds were classified as clean (50/50). The types of spinal procedures these patients underwent were as follows: 27/50 posterior instrumented fusion, 5/50 transforaminal lumbar interbody fusion (TLIF), 2/50 oblique lateral lumbar interbody fusion (OLIF), 1/50 anterior lumbar interbody fusion (ALIF), 4/50 posterior scoliosis correction, 4/50 insertion of growth rods, 4/50 removal of metalwork, and 3/50 revision instrumentation.

The mean duration for PICO dressing application was 6.68 days (SD = 5.88). In terms of the wound outcomes, none of the patients developed a seroma. Two out of the 50 (4%) patients developed a wound dehiscence and subsequent SSI. One of these was classified as superficial, with the other being a deep infection. Both were managed with antibiotics and re-application of PICO dressings. These resulted in the complete resolution of the SSI. None of these SSIs resulted in a return to theatre. The characteristics of this group of patients are summarized in Table 1.

**Table 1:** Measured outcomes for spinal surgery patients having PICO dressings postoperatively *Transforaminal lumbar interbody fusion; **Oblique lateral lumbar interbody fusion; ***Anterior lumbar interbody fusion

Age (mean, range)	32.7, 13-81 years
Gender (n,%)	
Males	30 (60%)
Females	20 (40%)
ASA Grade (n,%)	
1	30 (60%)
2	16 (32%)
3	4 (8%)
Wound classification (n,%)	
Clean	50 (100%)
Clean contaminated	0
Contaminated	0
Dirty	0
Types of Spinal Procedures (n,%)	
Posterior Instrumented Fusion	27 (54%)
TLIF*	5 (10%)
OLIF**	2 (4%)
ALIF***	1 (2%)
Posterior Scoliosis Correction	4 (8%)
Insertion of growth rods	4 (8%)
Removal of metalwork	4 (8%)
Revision Instrumentation	3 (6%)
Duration of PICO dressings (mean, SD)	6.68 days (SD=5.88)
Wound outcomes (n,%)	
Seroma	0
Dehiscence	2 (4%)
Surgical site infection	2 (4%) *Superficial = 1, Deep = 1*
Return to theatre (for SSI)	0

Data from the last six years for patients undergoing spinal surgery followed by standard dressing use show a mean of 800 ± 89 spinal procedures per annum, with a mean incidence of 9.27 ± 4.14 (1.15%) SSIs per annum. For the patients who developed SSIs, on average 77% of these required return to theatres. The data from each individual year with their subsequent SSI incidence rates are given in Table 2. 

**Table 2:** Comparative data for the incidence of surgical site infections for spinal patients during last six years * Number of patients who required a return to the theatre among the ones who developed surgical site infections following spinal surgery

	Spinal Procedures	Number of Surgical site infections	Incidence rate of Surgical Site Infections (%)	Number of Return to theatre *	Rates of return to theatre for SSI (%)
2016	938	14	1.49%	12	85.70%
2017	793	4	0.50%	3	75.00%
2018	690	6	0.86%	5	83.30%
2019	779	9	1.15%	5	55.50%
2020	707	15	2.12%	12	80.00%
2021	843	10	1.18%	10	100%
2022	880	13	1.48%	8	61.50%
PICO	50	2	4.00%	0	0%

The overall incidence of SSIs with PICO dressings compared to standard dressing use was not statistically significant (p = 0.087). Comparing the rates of return to theatres among the two dressing types used was also not statistically significant (p = 0.483). These are presented in Table 3.

**Table 3:** Statistical analysis SSI = Surgical site infections *Chi-square test (1 degree of freedom); ** Total number of spinal operations with regular dressings over six years

	PICO	Regular dressings	χ2-statistic*	p-value*
Total Operations	50	5630**	-	-
SSI	2	71	2.93	0.087
Return to theatre	0	55	0.49	0.48

We could not retrieve data for the subclassification of SSIs from previous years; therefore, these could not be compared.

## Discussion

Our study indicates that the use of PICO dressings as postoperative prophylaxis does not alter the incidence rate of SSIs in patients undergoing spinal surgery. The comparison between PICO and standard occlusive dressing use was not statistically significant.

The incidence of wound complications such as seroma or wound dehiscence was 0% for each respectively, which, although significant, was not comparable to the standard dressing use due to the lack of historical data on these parameters from previous years. We believe that this is an important result but, unfortunately, cannot be compared to test statistical significance.

Interestingly, for the two patients who did develop SSIs, their subsequent management did not require a return to theatres. Both were managed with a course of antibiotics along with re-application of PICO dressings for a longer duration of time. Although the return to theatres was zero in our sample population, our comparison group was quite large, and therefore the results were not statistically significant. Despite this, our fellow spinal surgeons believe that the use of PICO dressings following surgery holds the potential to reduce the rates of return to theatre among patients who develop SSIs following spinal procedures. This could mean that we can cut down a huge amount on the costs of managing these complications along with a reduction in morbidity and mortality.

Reviewing the literature indicates that the use of ciNPT across various surgical disciplines has been associated with favorable clinical outcomes [18]. Given the potential consequences and high costs of wound-related adverse events following spinal surgery, recent years have seen a trend to employing ciNPT for managing this patient population. Studies similar to this have compared ciNPT against regular dressings, reporting a lower incidence of SSIs in the former group [19-22].

A recent systematic review evaluating ciNPT use in spinal surgical patients established a statistically significant reduction in the incidence of SSIs [23] but did not highlight a reduction in the duration of hospital stay. Our study does highlight that ciNPT use can potentially reduce the duration of inpatient stays as fewer patients require a return to theaters.

Apart from clinical effectiveness, another important aspect to consider with regard to advocating a new treatment option is the associated cost and its feasibility. The use of ciNPT in open ventral hernia mesh repair [24], breast reconstruction [25], and vascular surgery [26] has proven cost-effective overall. More closely related to spinal surgery, revision knee arthroplasty surgery has also shown economic feasibility when ciNPT was employed [27].

The National Institute for Health and Care Excellence (NICE) recommendations on use of PICO dressings for closed surgical incisions estimate that PICO dressings offer an average of £101 per person when compared to standard dressings [16]. If we can find a relation between PICO dressing use and the reduction of the severity of SSIs occurring post-spinal surgery, this could drive the average cost savings per person even further, resulting in stronger evidence to support their routine use.

Our study was limited by the fact that our comparison patient data did not take into account patient factors such as age, type of procedure, ASA grades, and subclassifications of SSIs. Additionally, our patient population with the PICO dressings was small compared to the comparison group, which meant that any interesting results, which were not big enough in magnitude, would be statistically insignificant due to the large number in the comparison group.

Moving forward, we would recommend that further studies need to be undertaken in order to better evaluate the effectiveness of PICO compared with standard dressings. The literature provides strong evidence in terms of reduction in rates of SSI associated with PICO use in a wide spectrum of surgeries, but our study highlights an added potential benefit of minimizing the rates of return to theatres among spinal surgery patients developing SSIs.

## Conclusions

Our study reflects that there is no difference in the incidence rates for SSIs for patients who have PICO dressings versus those having standard occlusive dressings as postoperative closed surgical incision site wound closure following spinal surgery. For those who do develop SSIs, there was no difference in the rates of return to theatres among the two patient populations. Further studies with a larger number of patients are needed to establish a link between PICO dressing use and reduction in the incidence rates of SSIs and subsequent return to theatres for patients undergoing spinal surgery.
